# The Hospitals and the Insurance Scheme

**Published:** 1911-05-27

**Authors:** 


### The Hospital

A JOURNAL OF

The Medical Sciences and Hospital Administration.

NEW SERIES. NO. 225, VOL IX [NO. 1297, VOL. L.] SATURDAY, MAY 27, 1911

### THE HOSPITALS AND THE INSURANCE SCHEME.

LAST week we published the opinions of some of the hospital authorities with regard to the probable effects of the Government Insurance Scheme on the voluntary hospitals of this country. In this number we give some further excerpts from letters dealing with this subject, to which we desire to draw our readers' attention. The subject is so important, and it is so desirable that every side should be given a full opportunity of expressing its view, that no apology is needed for recurring to the matter and dealing with it in its various aspects.

The complicated nature of the Bill, as published, is such that it is at the present moment impossible to do more than forecast probabilities founded on a consideration of the clauses as they stand. No one can doubt that these clauses will be materially altered when the light of public discussion and debate has been thrown upon them, and we fully realise that the ultimate Act of Parliament which results from this Bill may be widely different from the draft measure now before the country. Yet it is with this draft measure and not with the final apotheosis of the Government scheme that we have to deal, and it is well that institutional workers should realise their responsibilities in the matter and make their opinions felt. We know of no better manner in which this may be done—done in such a way that the Government may be duly sensible of the weight of institutional opinion—than through the medium of a combined conference on the subject, as suggested by various correspondents in THE HOSPITAL. The British Hospitals Association is the proper organisation to initiate such a conference, and we sincerely trust that the officers of the Association will lose no time in arranging for the meeting. Our interests are too important, too far-reaching, and too valuable to be jeopardised by any mistaken modesty in giving due prominence to our considered opinion on a matter that affects them so vitally as this measure does. The Government has already announced its willingness to give careful attention to whatever objections may be brought against the scheme, and to take into consideration everything that may tend to make the ultimate solution of the problem of State insurance more effective. Admittedly not a party Bill to be criticised on narrow lines, but to be dealt with in a broad-minded spirit and with the determination that all criticism should be directed wholly to rendering the measure practicable and thorough, the Bill claims the most careful and yet the most searching investigation on the part of the institutional worker and the medical man in this country.

It shall be our object, in the columns of THE HOSPITAL, to give from time to time such information on the subject of State insurance and the hospitals in countries where similar schemes or modifications of them have been tried as may help our readers to obtain some data on which to base suggestions and put forward amendments, and we look to institutional workers to aid us in the endeavour to make these articles informative in the true sense of the word. The ventilation of opinions, especially when they are grounded on practical experience and a knowledge of the conditions which obtain in the hospital world, before such a conference as we have suggested takes place, will clear the ground to some extent and will enable those who are primarily concerned with the passage of the Bill through Parliament to grasp the significance of certain aspects of the measure which have not received the full attention which they undoubtedly merit. The medical profession, it is gratifying to note, is already bestirring itself, and in the course of the ensuing months it will doubtless deal *in extenso* with the provisions of the measure so far as these affect the practitioner. The Poor Law side, and that of the various co-operative societies, will be equally well cared for. But there remains the comparatively large aspect of the voluntary philanthropical interest to be considered in which the hospitals are primarily concerned. It is this side that needs special attention from the institutional worker. He alone can deal with it in a thoroughly knowledgeable manner, for he alone is in a position to judge, from his experience of, and his acquaintance with the conditions under which hospital work is carried on in this country, the probable effects of radical departures from the beaten track such as this measure foreshadows.

There will be many amateur critics who will, we may be sure, not neglect to meddle with the hospitals in their relation to the insurance scheme. Such critics will carefully collect the evidence that supports their own particular views and leave uncollated that which tends to vitiate their arguments, but, as is usual in such cases, they will lack no energy and power of voice to make their views widely known. In the circumstances there is, we fear, a real danger that valid objections to the measure, if they exist, may be disregarded, and provisions inimical to the best interests of the voluntary hospitals in this country introduced into the final scheme. To avert such a danger must be the object of every hospital worker, and success can only follow if the measure is criticised in a serious and calm manner, and if the opinions of the hospital world are expressed after that due reflection and with that necessary weight which concerted action by a representative body alone can give them. Already there is a tendency, even among the supporters of the Bill as it stands, to criticise the short space—some two days—within which the debate on the second reading is to be limited, and to press for at least another day for discussion. What is wanted is discussion in Committee by experts, and the most careful consideration first of principles and then of details. Such discussion must be premature until the opinions of those really qualified to advise shall have been ascertained.

